# Supplementary material for: Contact-free magnetic resonance imaging and spectroscopy with acoustic levitation
Source: Nat Commun. 2025 Apr 25;16:3917. doi: 10.1038/s41467-025-58949-2 (PMC12032270; doi:10.1038/s41467-025-58949-2)
Supplement: Supplementary file 1 — Supplementary Information [file 41467_2025_58949_MOESM1_ESM.pdf]

Supplementary Information

Contact-free magnetic resonance imaging and spectroscopy with acoustic levitation

Smaragda-Maria Argyri[1], Leo Svenningsson[1], Feryal Guerroudj[1], Diana Bernin[1], Lars Evenäs[1]*, and Romain Bordes[1]*

[1] Department of Chemistry and Chemical Engineering, Chalmers University of Technology, Gothenburg, Sweden

*: Corresponding author.

E-mail address: lars.evenas@chalmers.se

E-mail address: bordes@chalmers.se

Experimental set-up

Mechanical lift

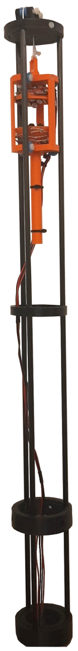

Figure S1: Photograph of the mechanical lift that was used for the transportation of the demagnetized acoustic levitator with the levitator sample in the MRI-bore.

Frequency response of acoustic levitator

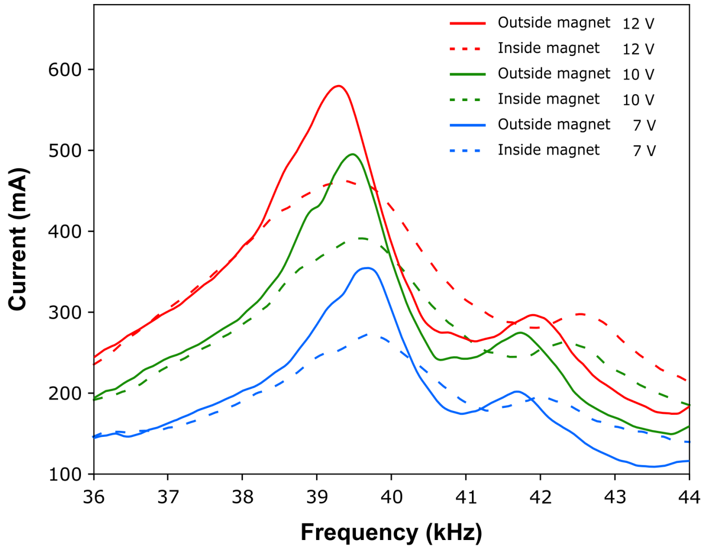

Figure S2: Frequency response of the demagnetized levitator inside (continuous lines) and outside (dashed lines) the magnet at 7 (lower, blue lines), 10 (middle, green lines), and 12 (upper, red lines) volt.

Simulations of acoustic pressure with respect to the temperature

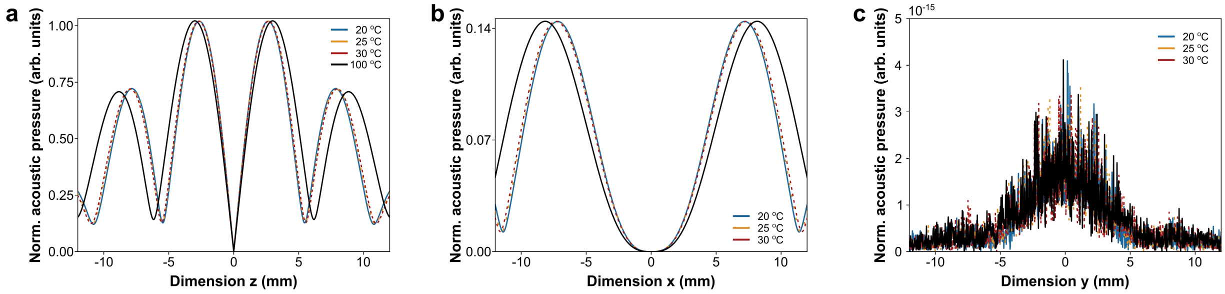

Figure S3: Normalized acoustic pressure of levitator Mk3 at 20 (blue), 25 (orange), and 30 °C (red) along the a) z, b) x, and c) y axis.

Magnetic resonance images

Droplet imaging and voxel selection

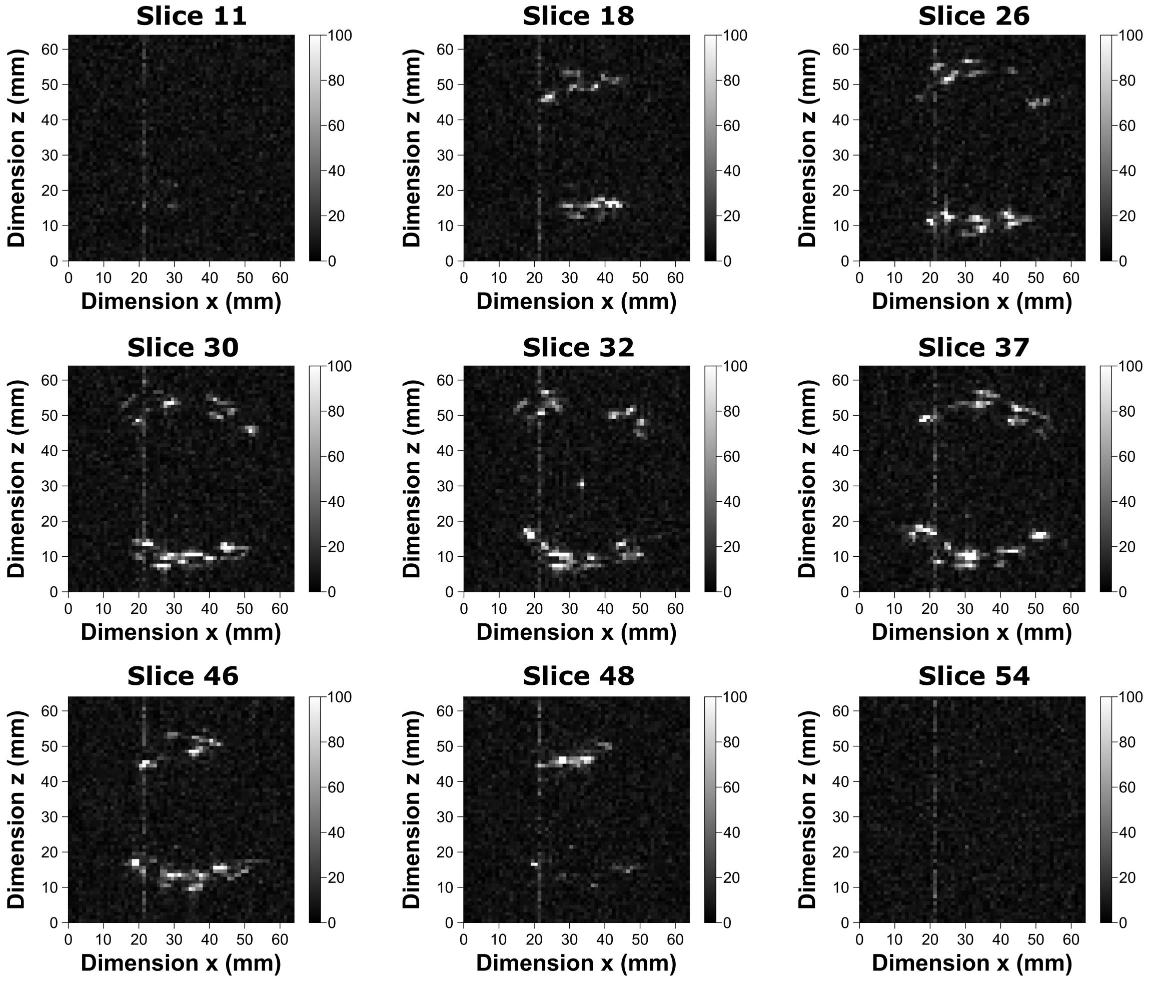

Figure S4: RARE MR images while a droplet of hexadecane was acoustically levitated at 9.0 V on different 1 mm thick slices along the sagittal (*xz*) plane where the transducers are visible too. We set a field of view 64x64 mm, ROI of 64x64 pixels, 8 averages, repetition time of 3000 ms, echo time of 2.28 ms, bp32 excitation and refocusing pulse, and RARE factor 1. The spherical microliter droplet with a radius of approximately 1 mm is visible only on the central magnetic resonance image (slice 32). The grayscale bar indicates the pixel intensity.

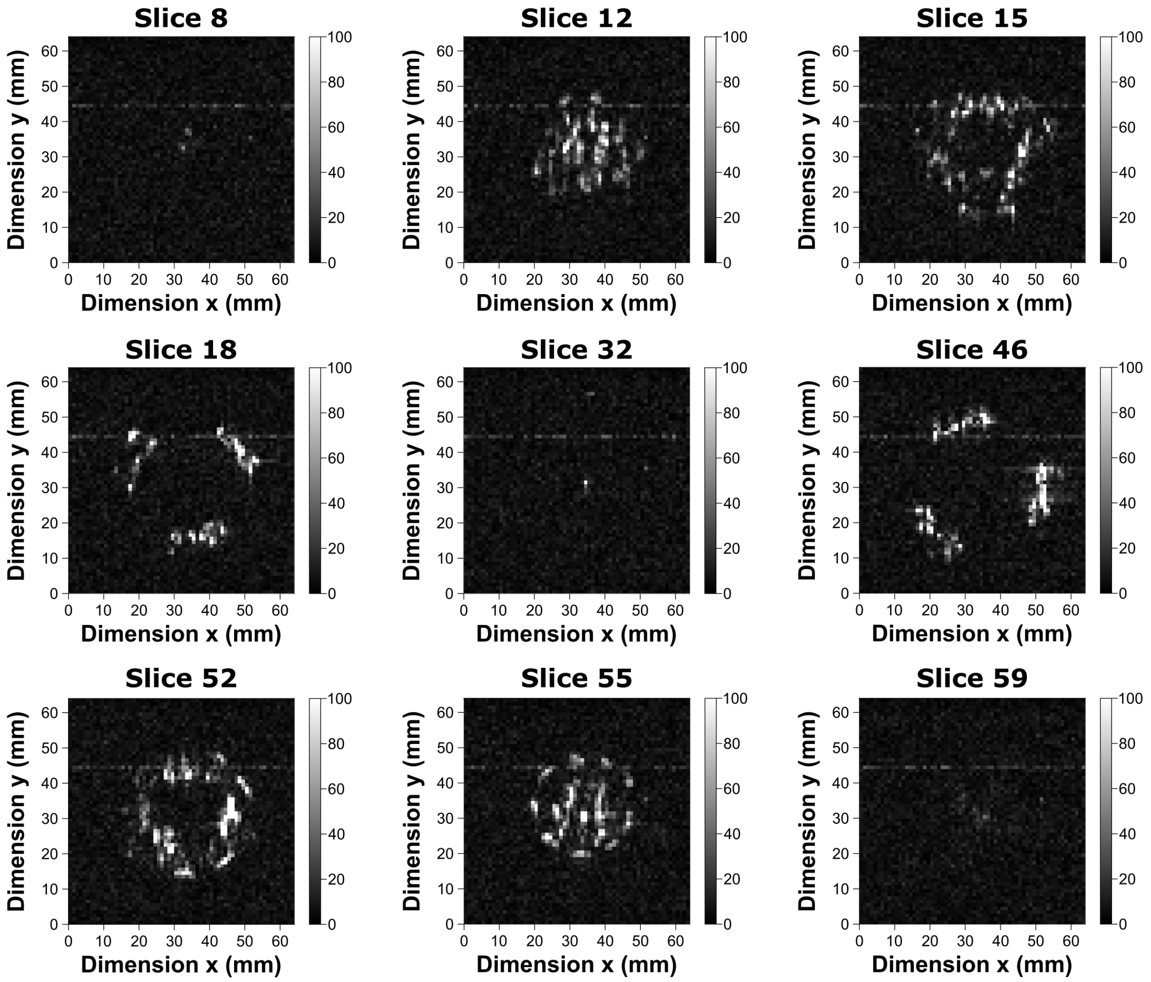

Figure S5: RARE MR images while a droplet of hexadecane was acoustically levitated at 9.0 V on different 1 mm thick slices along the axial (*xy*) plane where the transducers are visible too. We set a field of view 64x64 mm, ROI of 64x64 pixels, 8 averages, repetition time of 3000 ms, echo time of 2.28 ms, bp32 excitation and refocusing pulse, and RARE factor 1. The spherical microliter droplet with a radius of approximately 1 mm is visible only on the central magnetic resonance image (slice 32). The grayscale bar indicates the pixel intensity.

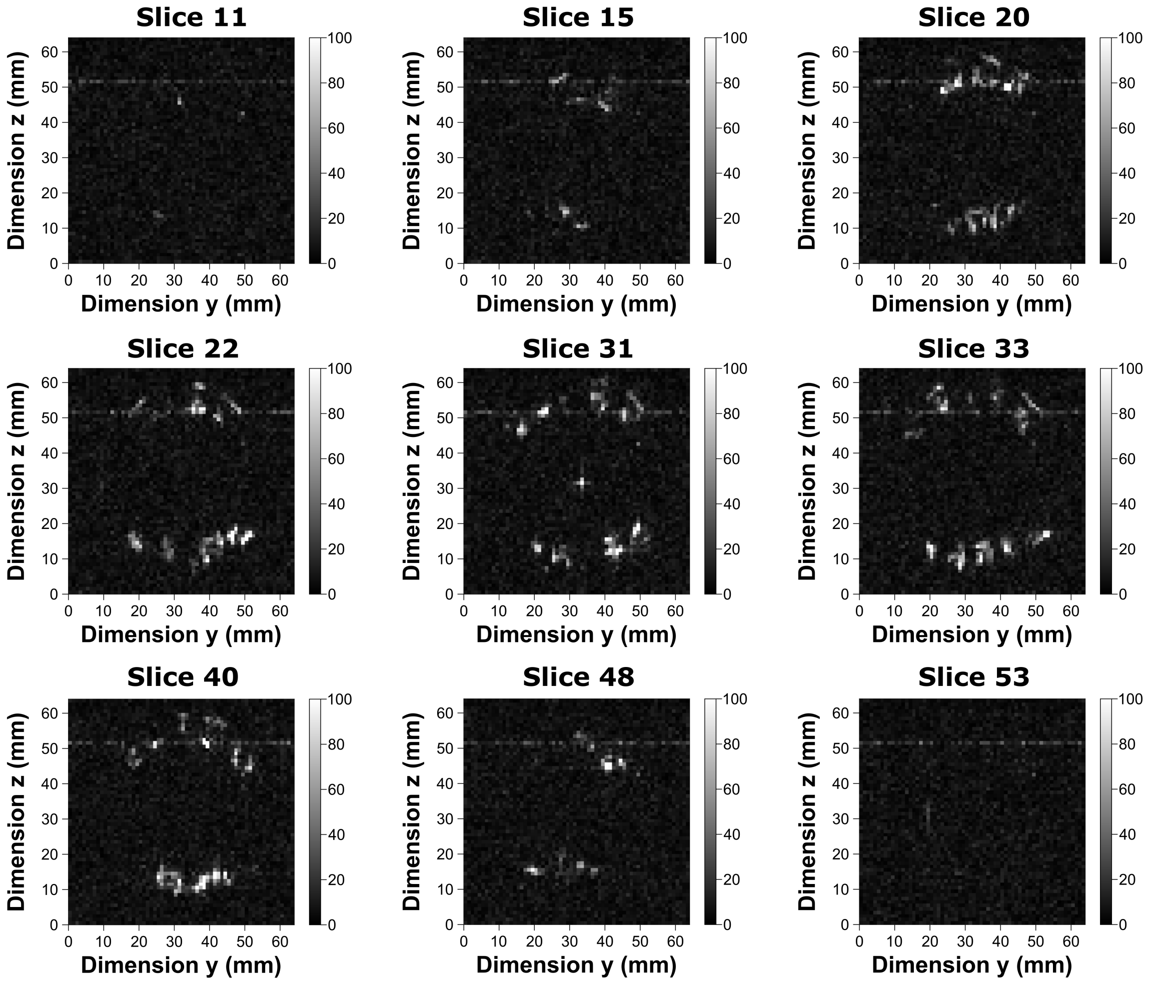

Figure S6: RARE MR images while a droplet of hexadecane was acoustically levitated at 9.0 V on different 1 mm thick slices along the coronal (*yz*) plane where the transducers are visible too. We set a field of view 64x64 mm, ROI of 64x64 pixels, 8 averages, repetition time of 3000 ms, echo time of 2.28 ms, bp32 excitation and refocusing pulse, and RARE factor 1. The spherical microliter droplet with a radius of approximately 1 mm is visible only on the central magnetic resonance image (slice 31). The grayscale bar indicates the pixel intensity.

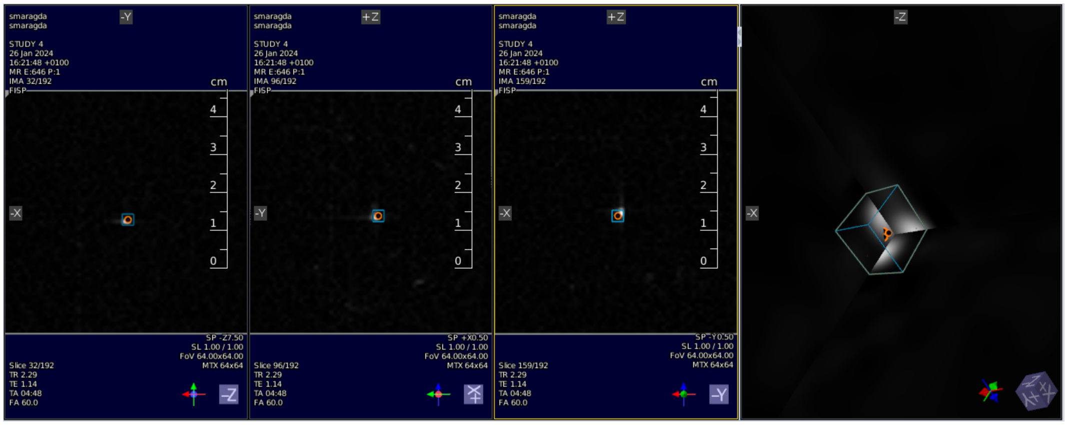

Figure S7: True-FISP MR images with a 3x3x3 mm voxel selections around an acoustically levitated droplet of canola oil at 9.0 V along the axial (*xy*), sagittal (*xz*) and coronal (*yz*) planes, and 3D intersection of the slices depicting the droplet. We set a field of view 64x64 mm, ROI of 64x64 pixels, 1 average, repetition time of 2.29 ms, echo time of 1.14 ms.

Exploding droplet

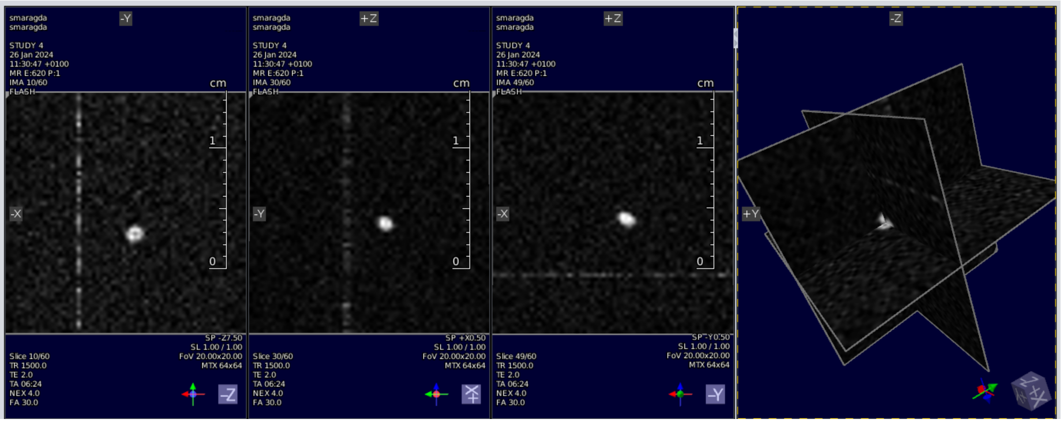

Figure S8: FLASH MR images of an acoustically levitated droplet of hexadecane at 9.0 V along the axial (*xy*), sagittal (*xz*) and coronal (*yz*) planes, and 3D intersection of the slices depicting the droplet. We set a field of view 20x20 mm, ROI of 64x64 pixels, 4 averages, repetition time of 1500 ms, echo time of 2.0 ms, bp32 excitation pulse.

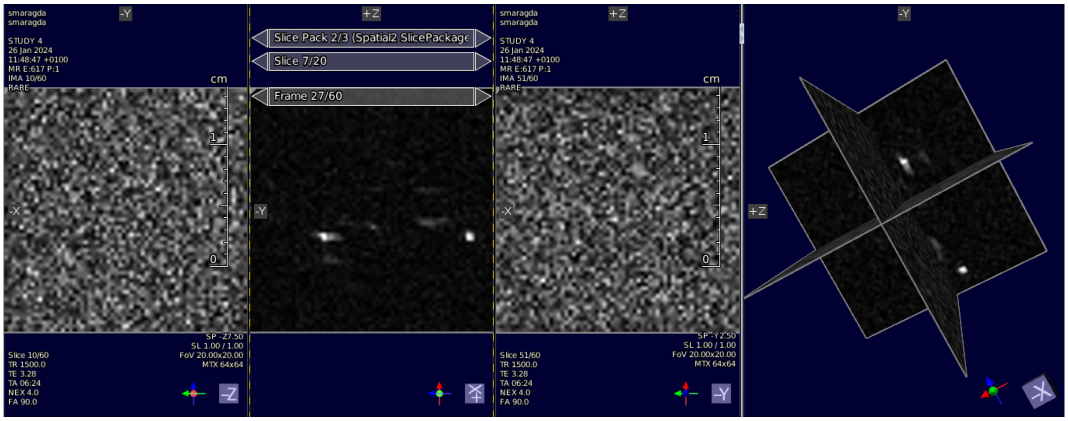

Figure S9: RARE MR images of the acoustically levitated droplet of hexadecane at 9.0 V that was previously imaged with FLASH in Fig. S8, while being burst along the sagittal (*xz*) plane. No signal was recorded along the axial (*xy*), and coronal (*yz*) planes. In the last subfigure on the right, a 3D intersection of the slices is depicted. We set a field of view 20x20 mm, ROI of 64x64 pixels, 4 averages, repetition time of 1500 ms, echo time of 3.28 ms, calculated excitation, and refocusing function, and RARE factor 1.

FLASH MR images of evaporating water droplet

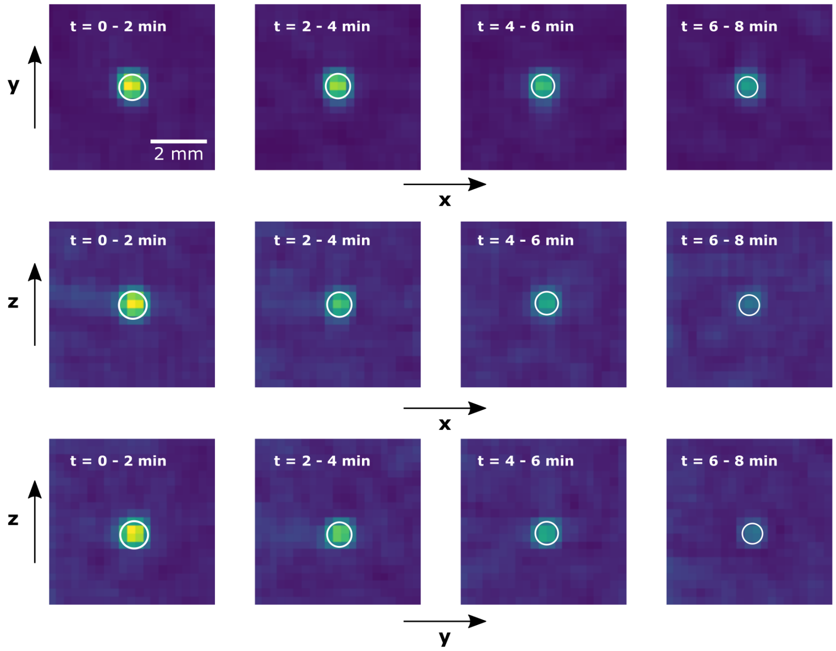

Figure S10: FLASH MR images of an acoustically levitated droplet of milli-q water, at 10 V, along the axial (*xy*), sagittal (*xz*), and coronal (*yz*) planes, with white overlying fittings of the droplet contours, over a period of approximately 8.5 min. The time in the inset refers to the period of acquisition.

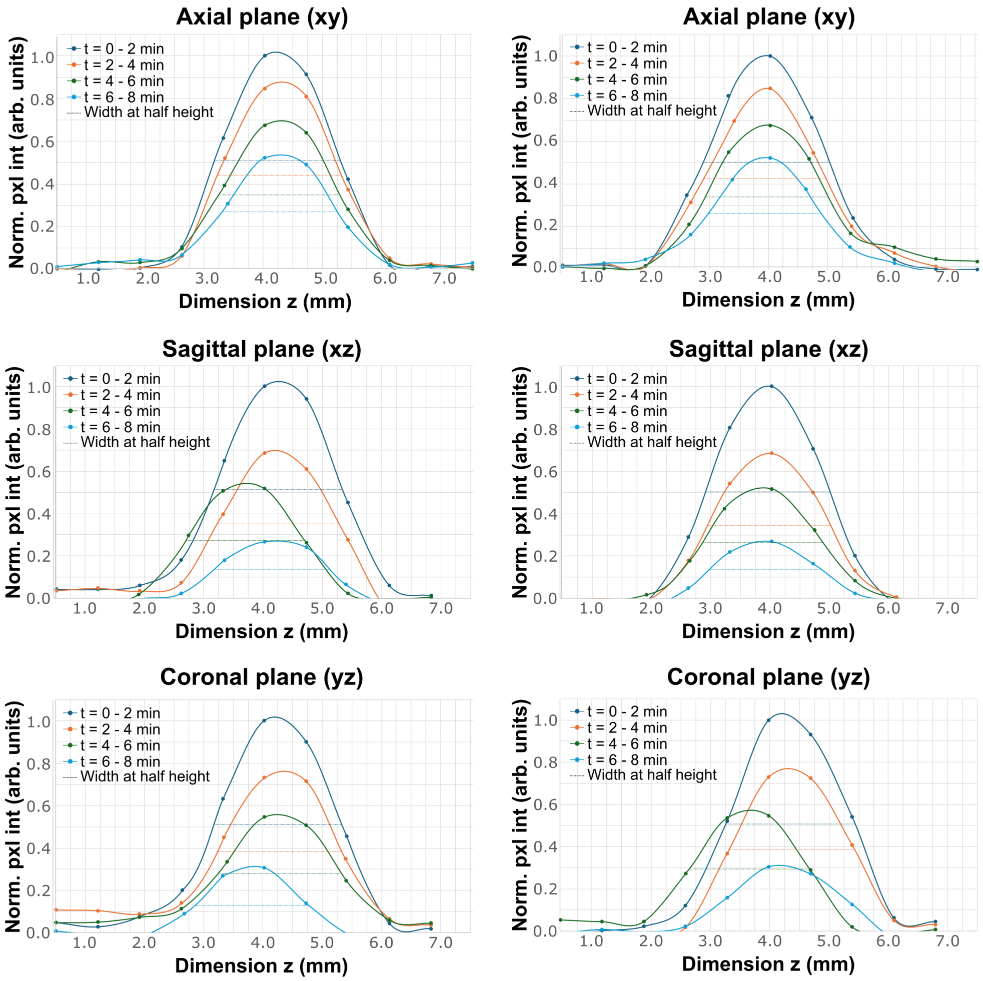

Figure S11: Normalized pixel intensity around the levitated droplet of water, with respect to the axial distance, from the 4 MR images that were acquired over a period of 8.5 min. The width at half height was estimated, from which we calculated the volume and aspect ratio of the droplet, over time.

The average widths at half the height of the milli-q water droplet are shown in Table S1:

Table S1: Average diameters along the x, y, and z dimension of the water droplet, at each repetition, estimated from the width at half height in Fig. S11.

Dimension (mm)	Repetition 1	Repetition 2	Repetition 3	Repetition 4
x	2.15	2.08	2.03	1.95
y	2.15	2.08	2.08	1.91
z	2.13	2.10	2.05	1.94

From the values shown in Table S1, we calculated the average x, y, and z values,

and then determined the volume from the equation:

$$V_{\text{obl}} = \frac{4}{3} \cdot \pi \cdot a \cdot b \cdot c \tag{S1}$$

where, a, b, c are the radii of the droplet along the x, y, and z axial dimensions, respectively.

True-FISP MR images of water evaporation

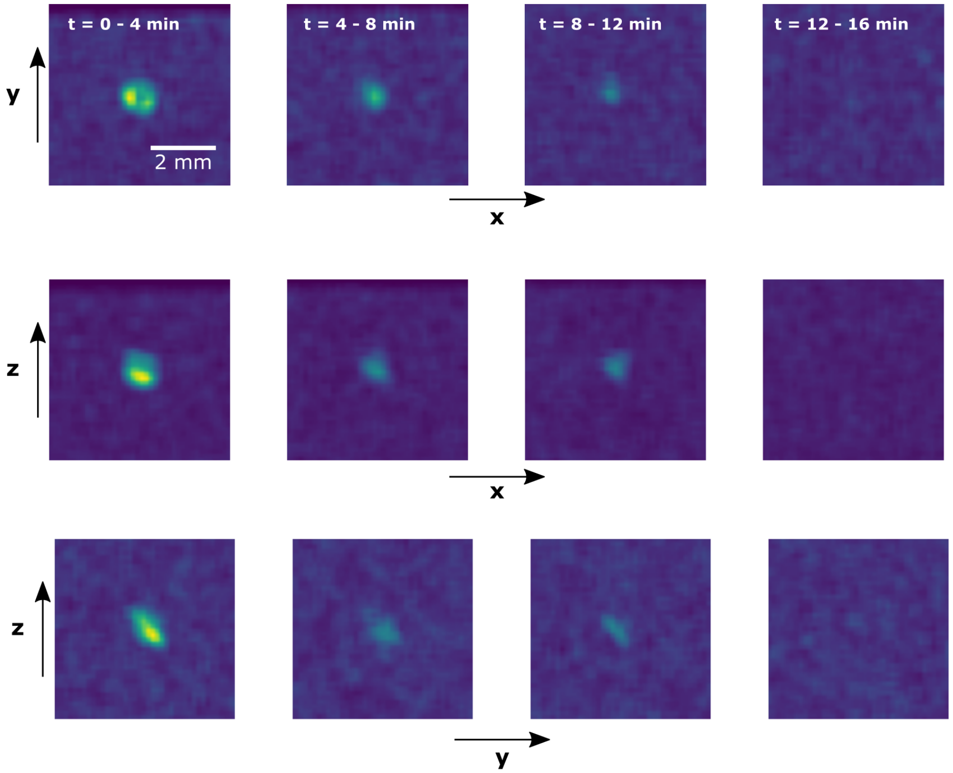

Figure S12: True-FISP MR images of an acoustically levitated water droplet at 10.0 V along the axial (xy), sagittal (xz), and coronal (yz) planes. We set 32x32 mm field of view, 128x128 pixel ROI, 1 mm slices thickness, 1 average, echo time of 1.783 ms, repetition time of 3.567 ms, 64 segments, 32 slices, bp32 excitation pulse, and applied 4 repetitions with a total scan time of 16 min and 25 sec. The images were plotted with Python. The time in the inset refers to the period of acquisition.

True-Fisp MR images of hexadecane at different voltages

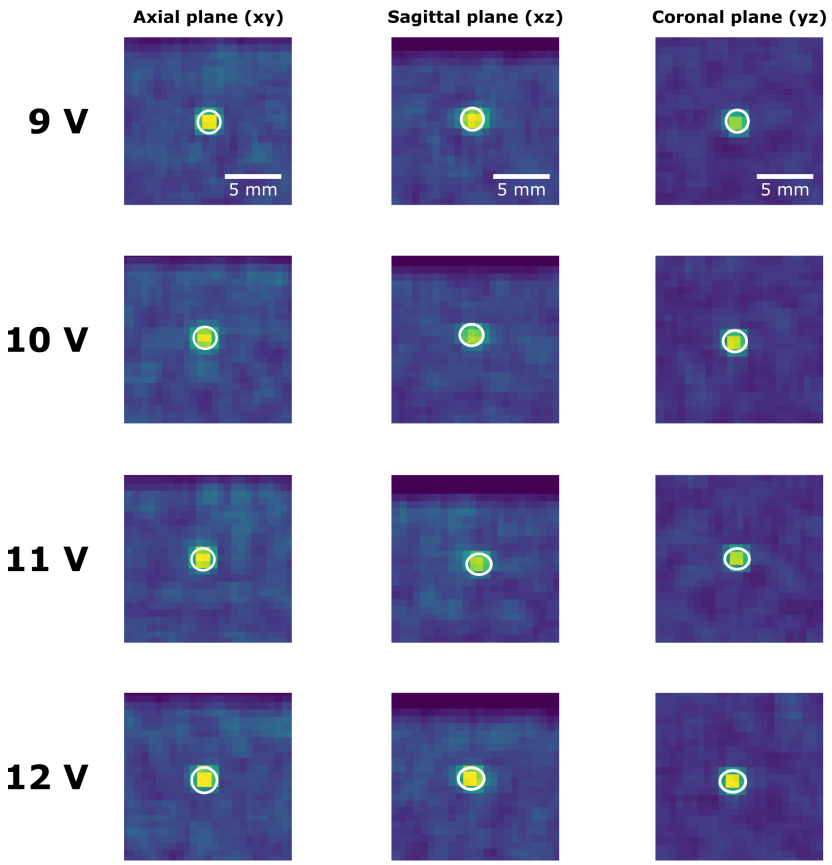

Figure S13: True-FISP MR images of an acoustically levitated droplet of hexadecane at 9.0 V, 10.0 V, 11.0 V, and 12.0 V, along the axial (*xy*), sagittal (*xz*), and coronal (*yz*) planes, with white overlying fittings of the droplet contours. We set 40x40 mm field of view, 64x64 pixel ROI, 1 mm slices thickness, 1.84 ms for echo time, and 1000 ms for repetition time. The images were plotted with Python and adjusted by applying a 3x3 kernel with values of 0.1.

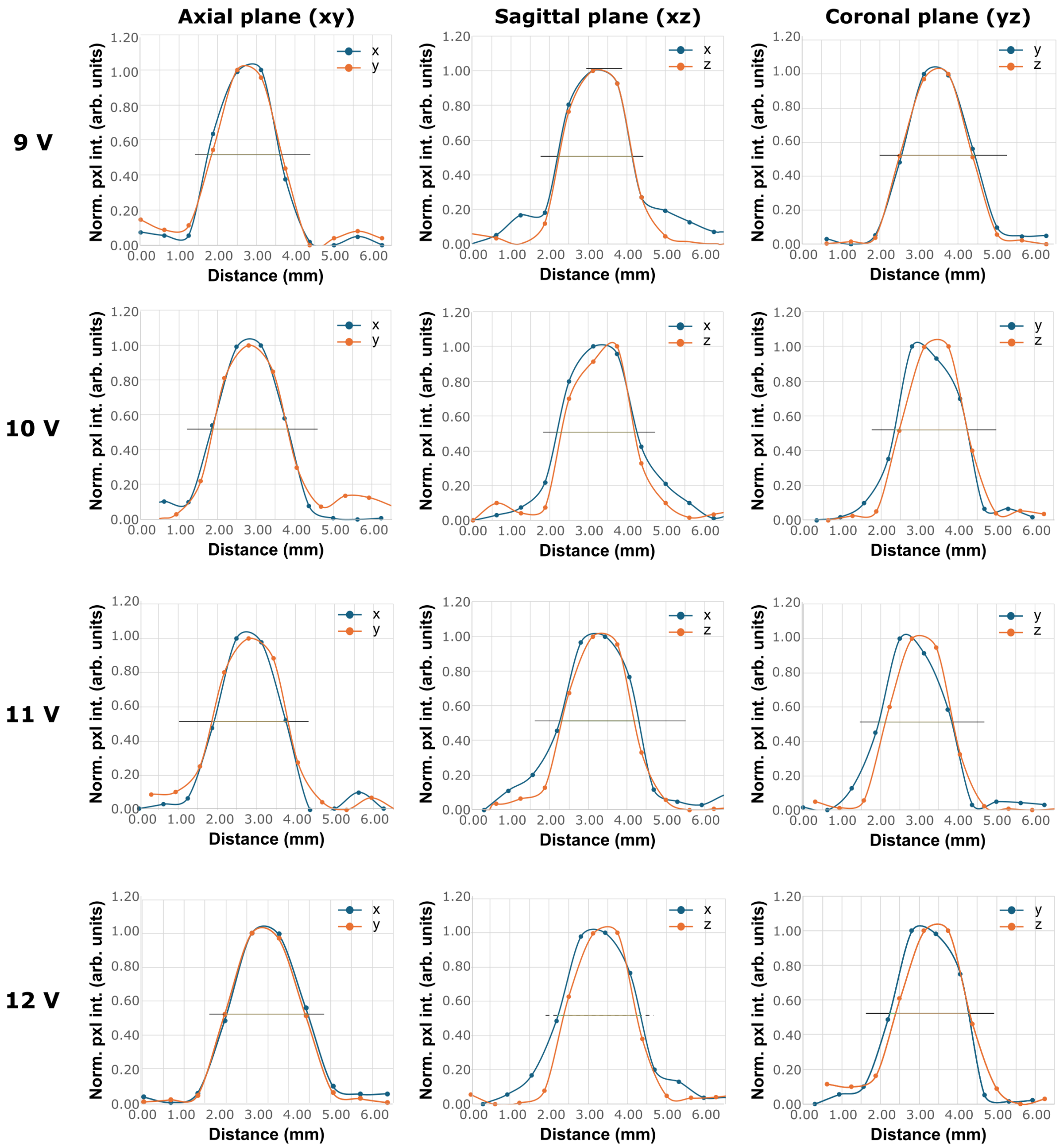

Figure S14: Normalized pixel intensity around the levitated droplet of hexadecane at different voltages, with respect to the axial distance, along the axial (*xy*), sagittal (*xz*), and coronal (*yz*) planes. The width at half height was determined, from which we calculated the aspect ratio of the droplet.

The average widths at half the height of the hexadecane droplet are shown in Table S2:

From the values presented above and Eq. (S1) we calculated the volume of the droplet to be approximately 3.75 μL.

Nuclear magnetic resonance spectra

Canola oil MR spectra

Table S2: Average diameters along the x, y, and z dimension of the hexadecane droplet, levitated at different voltages, estimated from the width at half height in Fig. S14.

Voltage (V)	x (mm)	y (mm)	z (mm)
9	1.90	1.85	1.89
10	2.02	1.90	1.83
11	1.95	1.92	1.81
12	2.10	2.06	1.90

Table S3: Chemical shifts of signals in ppm, of the MR spectra recorded with the localized pulse sequences STEAM, PRESS, and ISIS, together with the non-localized pulse sequences NSPECT and Single Pulse on an acoustically levitated droplet of canola oil at 9.0 V, in a 7.05 T magnet with a 66 mm diameter MRI probe, compared with the spectrum recorded with a Single Pulse on canola oil in a glass tube, in a 7.05 T magnet with a 5 mm NMR probe.

STEAM (droplet)	PRESS (droplet)	ISIS (droplet)	NSPECT (droplet)	Single Pulse (droplet)	Single Pulse (glass tube)
-	5.53	5.52	5.52	5.53	5.52
-	4.48	4.47	4.47	4.47	4.47
-	4.29	4.30	4.29	4.29	4.29
2.96	2.96	2.98	2.96	2.96	2.97
2.45	2.44	2.44	2.45	2.45	2.45
2.25	2.24	2.23	2.24	2.24	2.23
1.80	1.78	1.79	1.79	1.79	1.79
1.52	1.51	1.51	1.52	1.52	1.51
-	-	-	1.49	1.49	1.49
1.09	1.09	1.10	1.09	1.09	1.10

Table S4: Line widths at half height of signals in Hz, of the MR spectra recorded with the localized pulse sequences STEAM, PRESS, and ISIS, together with the non-localized pulse sequences NSPECT and Single Pulse on an acoustically levitated droplet of canola oil at 9.0 V, in a 7.05 T magnet with a 66 mm diameter MRI probe, compared with the spectrum recorded with a Single Pulse on canola oil in a glass tube, in a 7.05 T magnet with a 5 mm NMR probe.

STEAM (droplet)	PRESS (droplet)	ISIS (droplet)	NSPECT (droplet)	Single Pulse (droplet)	Single Pulse (glass tube)
-	15.63	12.67	10.9	5.55	12.45
-	19.78	13.75	4.48	7.20	6.65
-	16.37	15.24	4.48	5.92	6.04
20.55	14.31	16.53	6.31	6.28	12.60
29.35	8.47	9.74	5.76	5.73	6.96
22.38	23.29	20.38	7.23	7.02	13.97
19.81	21.27	18.73	20.62	18.06	20.84
40.66	7.76	20.88	14.16	14.34	14.71
-	-	-	8.98	9.00	8.14
11.38	10.27	11.02	5.95	5.73	6.50

Series of hexadecane ISIS MR spectra

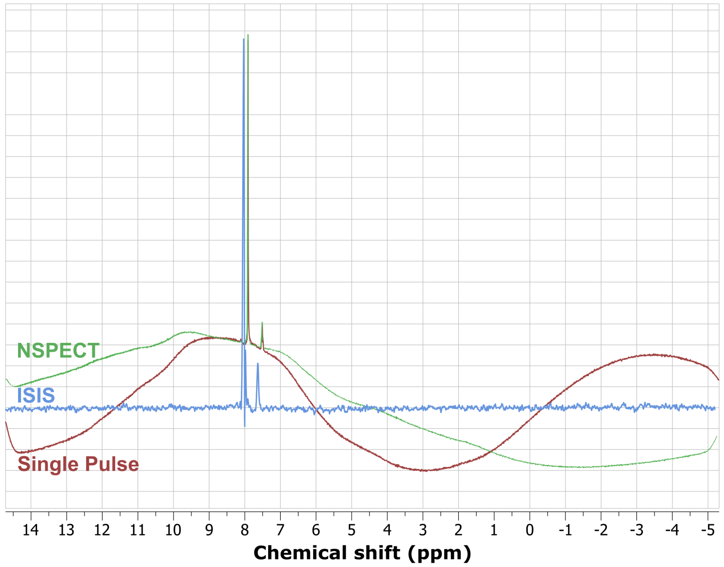

Figure S15: Baseline comparison between NSPECT (green), ISIS (blue), and Single Pulse (red) of an acoustically levitated hexadecane droplet at 9.0 V. For the pulse sequence ISIS we applied 8 averages (64 ISIS averages), 1500 ms repetition time, on a 3x3x3 mm voxel. For NSPECT and Single Pulse, we applied 40 and 20 averages, respectively, and 1500 ms repetition time. The scan times were 40 sec and 20 sec, with NSPECT and Single Pulse, respectively. The phase of the spectrum collected with ISIS was manually corrected, and no further adjustments were made.

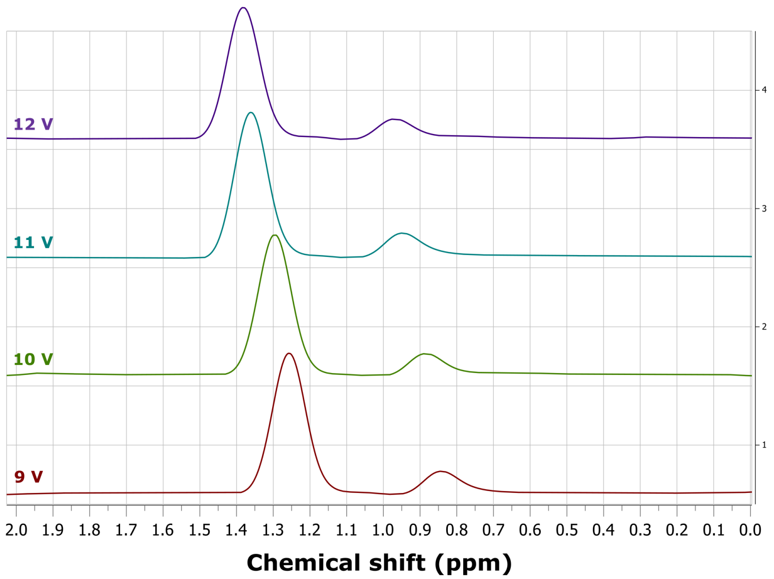

Figure S16: ISIS MR spectra of an acoustically levitated hexadecane droplet at 9.0 V, 10.0 V, 11.0 V, and 12.0 V. We applied 8 averages (64 ISIS averages), 1500 ms repetition time, on a 3x3x3 mm voxel. We applied 5 Hz exponential line broadening, the phase was manually corrected, and the baselines were corrected by applying Bernstein polynomial fit with an order of 3.

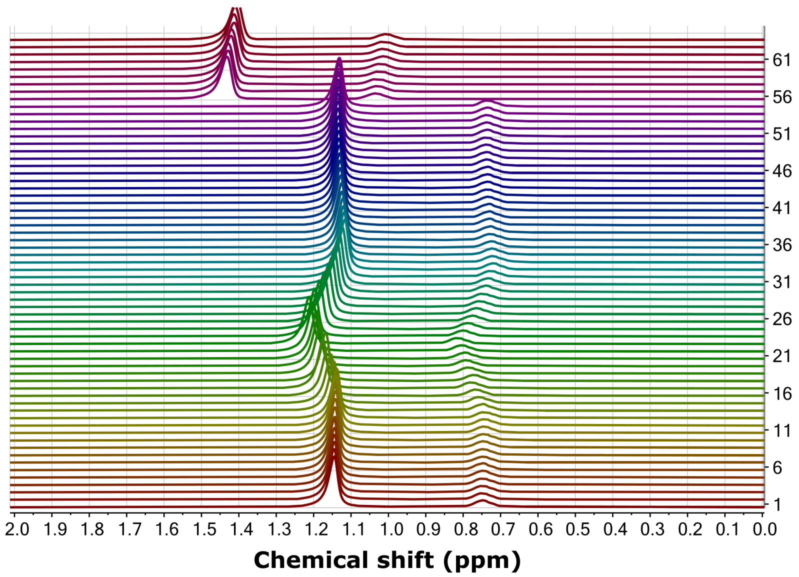

Figure S17: Series of ISIS MR spectra of an acoustically levitated hexadecane droplet with (from bottom to top) 15 spectra at 9.0 V, 10 spectra with a voltage increase from 9.0 V to 10.0 V with a step of 0.1 V, 10 spectra with a voltage decrease from 10.0 V to 9.0 V with a step of 0.1 V, 20 spectra at 9.0 V, and 7 spectra at 12.0 V. The spectra were acquired by setting 8 averages (64 ISIS averages), 1500 ms repetition time, on a 3x3x3 mm voxel. We applied 5 Hz exponential line broadening, the phase was manually corrected, and the baselines were corrected by applying Bernstein polynomial fit with an order of 3.

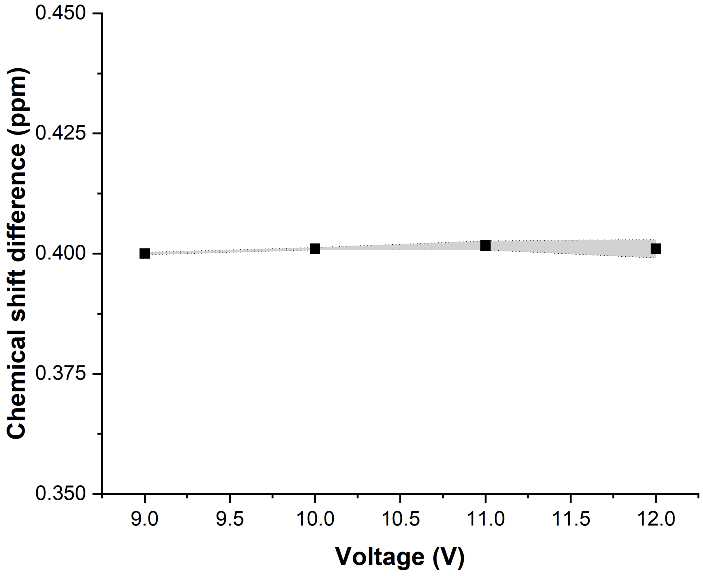

Figure S18: Chemical shift differences between the signals of hexadecane with respect to the applied voltage. The increased error with voltage is most likely related to a steady-state heating dependence from the pulses. The measurements were repeated a minimum of 3 times and the error was calculated from the standard deviation.

Droplet shape and chemical shift

In response to an external magnetic field B_0, the nuclear spins of the nuclei align parallel or antiparallel to the field, followed by the precession around the direction of the field. The resonance frequency of the nuclei, ω_0, is related to the effective magnetic field experienced by the nuclei, B_{eff}, according to the Larmor equation:

$$\omega_0 = \gamma B_{\text{eff}} \tag{S2}$$

where, γ, is the gyromagnetic ratio of the nucleus.

The effective magnetic field depends on the external magnetic field, B_0, permeability, μ, and local magnetic field perturbations present in the sample. Perturbations arise from demagnetizing effects related to the volumetric magnetic sus-

ceptibility, χ_v, and geometric parameters of the sample that are expressed by the geometric demagnetization factor, N.

For linear and homogeneous materials, the magnetic field within spheres and oblates is uniform [1]. In this case, the exact solution to the effective magnetic field in a spherical or oblate droplet surrounded by vacuum is written in scalar form as:

$$H_0 = \frac{B_0}{\mu_0} \tag{S3}$$

$$M = \frac{\chi_{\mathrm{v}} H_0}{1 + \chi_{\mathrm{v}} N} \tag{S4}$$

$$B_{\mathrm{eff}} = \mu_0 \left(1 + \chi_{\mathrm{v}}\right) \left(H_0 - NM\right) = \mu \left(H_0 - NM\right) \tag{S5}$$

where, H_0 is the magnetic field strength, B_0 is the magnetic field flux in the z-direction, μ_0 is the magnetic permeability of vacuum, and M is the magnetization [2, 3, 4].

The relative magnetic permeability, μ_{r}, relates to the volumetric susceptibility, χ_{v}, according to the equation [4]:

$$\mu_{\mathrm{r}} = \chi_{\mathrm{v}} + 1 \tag{S6}$$

$$\mu_{\mathrm{r}} = \frac{\mu}{\mu_0} \tag{S7}$$

with, μ_0 being the magnetic permeability of vacuum. Hence, the magnetic permeability can be expressed as:

$$\mu = (\chi_{\mathrm{v}} + 1)\mu_0 \tag{S8}$$

where, the volumetric susceptibility, χ_{v}, is expressed in SI units as:

$$\chi_v = \chi \cdot d \cdot \mathrm{MW} \cdot 4\pi \tag{S9}$$

where, χ is the susceptibility, d the density, and MW the molecular weight.

The droplet shapes we explored were the sphere and the oblate ellipsoid with the flat side aligned with the B_0 field and aspect ratio in the range of 0.200 to 0.999.

The geometric demagnetization factor, N for a sphere is $1/3$, which simplifies Eq. (S5) to Eq. (S11):

$$B_{\text{eff, sph}} = \frac{3\mu_0 (1 + \chi_{\mathrm{v}})}{\mu_0 (1 + \chi_{\mathrm{v}}) + 2\mu_0} \tag{S10}$$

$$B_0 = \frac{3\mu}{\mu + 2\mu_0} B_0 \tag{S11}$$

The description of the geometric demagnetization factor, N, for an oblate is given by Shine and Armstrong [5], in Eq. (S12)-(S14):

$$N_{\text{oblate}} = 1 - A \tag{S12}$$

$$A = \frac{d\cos^{-1} d}{(1-d^2)^{3/2}} - \frac{d^2}{1-d^2} \tag{S13}$$

$$d = \frac{a}{b} \tag{S14}$$

The constants a and b are the ellipsoid axis lengths as shown in Fig.2b with $a < b$. Hence, from Eq. (S5) the effective field for an oblate is expressed as:

$$B_{\text{eff, obl}} = \mu \left[H_0 - \left(1 - \frac{d\cos^{-1} d}{(1-d^2)^{3/2}} - \frac{d^2}{1-d^2} \right) M \right] \tag{S15}$$

Since acoustic levitation cannot occur in a vacuum, the equations are also adjusted for the relative magnetic permeability in air. The magnetic resonance frequency is proportional to the magnetic field (Eq. (S2)), and the resulting resonance frequency is:

$$\omega_{\text{drop}} = \frac{B_{\text{eff, drop}} - B_0}{B_0} \omega_0 \tag{S16}$$

Then, the chemical shift of the droplet is given by:

$$\delta_{\text{drop}} = \frac{\omega_{\text{drop}} - \omega_{\text{ref}}}{\omega_{\text{ref}}} \cdot 10^6 \tag{S17}$$

where, ω_{ref} is the reference frequency of the magnet.

In standard magnetic resonance studies, reference compounds are used for the chemical shift calibration of the MR spectra. In this study, we levitated a droplet

at low voltage and used MR imaging to confirm the sphericity of the droplet. Then, we acquired an MR spectrum which was shifted compared to where it would have appeared if tetramethylsilane (TMS) was used as a reference compound. For simplicity, we set the chemical shift of the highest peak at the shift where it would appear under TMS calibration. Then, we defined the chemical shift difference from a spherical to an oblate droplet as:

$$\delta_{\text{diff}} = \delta_{\text{drop, obl}} - \delta_{\text{drop, sph}} \tag{S18}$$

Evaporation of a 50 wt% aqueous TEG droplet

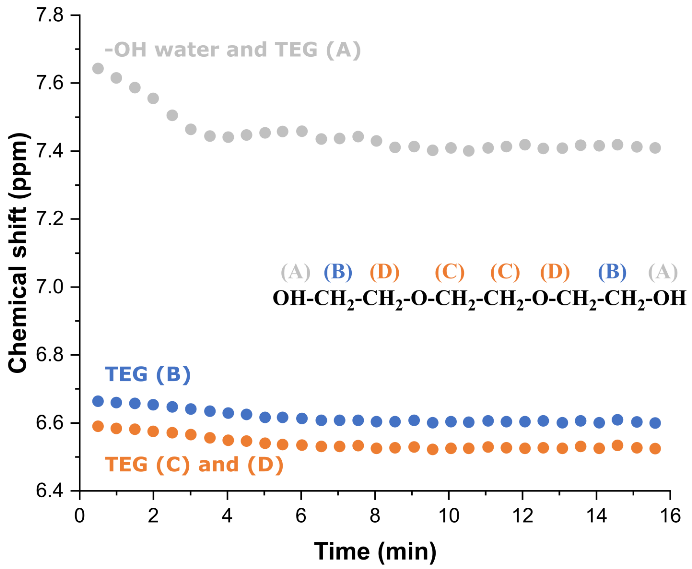

Figure S19: Chemical shifts of signals present in the 50 wt% TEG solution of the acoustically levitated droplet over time.

References

[1] Osborn, J. A. Demagnetizing factors of the general ellipsoid. *Phys. Rev.* **67**, 351, DOI: https://doi.org/10.1103/PhysRev.67.351 (1945).

[2] Jackson, J. D. (ed.) *Classical electrodynamics* (Wiley, New York, NY, 1999), 3 edn.

[3] Nayfeh, M. H. & Brussel, M. K. (eds.) *Electricity and Magnetism* (Wiley, New York, NY, 1985).

[4] Prozorov, R. & Kogan, V. G. Effective demagnetizing factors of diamagnetic samples of various shapes. *Phys. Rev. Appl.* **10**, 014030, DOI: https://doi.org/10.1103/PhysRevApplied.10.014030 (2018).

[5] Shine, A. D. & Armstrong, R. C. The rotation of a suspended axisymmetric ellipsoid in a magnetic field. *Rheol. Acta* **26**, 152–161, DOI: https://doi.org/10.1007/BF01331973 (1987).
